# Effects of Occlusal Hypofunction on Osteoclastogenesis Induced by Periodontal Ligament Cells

**DOI:** 10.1155/ijod/9926168

**Published:** 2025-10-14

**Authors:** Tomomi Takahashi, Tadashi Iizuka

**Affiliations:** Support Section for Education and Research, Faculty of Dental Medicine, Hokkaido University, Sapporo, Japan

## Abstract

**Background:**

Periodontal ligament (PDL) cells express several osteoclastic cytokines, including receptor activator of nuclear factor kappa ligand (RANKL) and osteoprotegerin (OPG). They regulate the alveolar bone structure by influencing osteoclast activity. The structure of the PDL is affected by mechanical stress. However, few in vitro studies have examined the effects of hypofunctional occlusion in isolated PDL cells. This study aimed to elucidate the role of PDL cells in osteoclastogenesis under hypofunctional occlusal conditions.

**Methods:**

Left maxillary molars were extracted to eliminate the occlusal force. Three weeks later, the histology and cytology of the PDL tissue samples were analyzed. To investigate osteoclast formation, isolated PDL cells were cocultured with RAW 264.7 cells.

**Results:**

The PDL tissue demonstrated atrophic changes on the non-occlusal side (N-Oc side). Immunocytochemistry and western blotting indicated that the isolated PDL cells were positive for RANKL and OPG; however, OPG expression was lower on the N-Oc side compared with the occlusal side (Oc side). Coculturing of PDL and RAW 264.7 induced the formation of osteoclast-like cells in all experimental groups. The N-Oc side exhibited more and larger osteoclasts than the Oc side. Osteoclast-like cells supplemented with RANKL and anti-OPG antibodies were significantly larger than those supplemented with RANKL alone.

**Conclusions:**

These findings show that the biological functions of PDL cells are altered by occlusal hypofunction and that a reduction in OPG expression promotes osteoclast differentiation.

## 1. Introduction

The periodontal ligament (PDL) is a group of specialized connective tissue fibers that attach teeth to the alveolar bone. PDL cells exhibit alkaline phosphatase activity [1, 2] and express osteopontin [3] and osteocalcin [2, 4], both of which are involved in bone formation. They also express several osteoclastic cytokines such as receptor activator of nuclear factor kappa ligand (RANKL) and osteoprotegerin (OPG) [5–8]. These cells regulate alveolar bone structure by influencing osteoclast activity.

As a source of mechanical stress in the oral region, occlusal stimuli, such as chewing food, exert great forces on periodontal tissue. The occlusal forces applied to the teeth are transferred to the alveolar bone and PDL, which are then subjected to mechanical stress. These forces contribute to the maintenance of functional PDL structures, and normal occlusion is essential for periodontal tissue homeostasis.

Periodontal tissue is affected by traumatic occlusion or orthodontic forces. Histopathological evaluation of experimental traumatic occlusion has shown an increased number of osteoclasts around the root and cementum resorption in the apex [9, 10]. Orthodontic tooth movement results from the resorption and formation of the alveolar bone. Osteoclastic activity on the compression side and osteoblastic activity on the tension side are seen during tooth movement [11, 12]. The expression of osteoclastic and osteoblastic factors is influenced by strong acute forces. Further, recent studies have suggested that microRNAs (miRNAs) are involved in bone remodeling in orthodontics [13].

Some studies have reported that the alveolar bone and PDL are altered by hypofunctional occlusions. They observed atrophic PDL cells, reduction in alveolar bone volume, root changes, and an increase in microvascular structures in the periodontal area. The loss of occlusal stimulation induces histological changes in the PDL [14, 15]. In such cases, there is a possibility that the biological functions of PDL cells may decline. Few studies have investigated the relationship between the phase of PDL cells and osteoclastogenesis following the loss of occlusal stimuli [16]. Furthermore, no in vitro studies have examined the effects of hypofunctional occlusion in isolated PDL cells. Therefore, the morphological responses to the loss of occlusal stimulation and the associated changes in cell biological functions have yet to be evaluated.

This study aimed to elucidate the role of PDL cells in osteoclastogenesis under hypofunctional occlusal conditions.

## 2. Materials and Methods

### 2.1. Animals

This study was performed with 20 4-week-old male Wistar rats (4 for histology and 16 for cytobiology) obtained from Japan SLC. Rats were maintained under controlled laboratory conditions (ambient temperature 23–25°C, relative air humidity 50%–60%, 12-h light/12-h dark cycle with light on at 7:00 a.m.) and were allowed to acclimate to their cages for 1 week after arrival. They were housed in a plastic cage (four rats per cage) with a soft animal bed and were fed a standard pellet diet with tap water ad libitum. All animal experimental procedures were performed between 9:00 and 12:00 a.m. in a dedicated animal laboratory. All animal experimental protocols were approved by the Institutional Animal Care and Use Committee of Hokkaido University (Number 22-0069) and were performed in accordance with the Animal Experimental Guidelines. This study corroborates the ARRIVE guideline for the reporting of animal studies.

### 2.2. Experimental Design

Five-week-old male Wistar rats (weighing 100–120 g) were used in the present study. The rats were sedated with a combination of isoflurane inhalation and intraperitoneal injection of three anesthetic agents (0.75 mg/kg medetomidine, 2 mg/kg midazolam, and 2.5 mg/kg butorphanol). The animals were placed on the operating table to facilitate dental treatment. To eliminate the occlusal force on the mandibular left molars, the first, second, and third left maxillary molars were extracted. After the extractions, animals received pain relief medication to minimize suffering. The rats were euthanized 3 weeks after surgery by overdose of isoflurane inhalation anesthesia. The histology and cytobiology of PDL tissue samples were analyzed.

### 2.3. Histopathology

The mandibles of the rats were removed and immediately fixed in 10% neutral-buffered formalin; they were demineralized in 10% ethylenediaminetetraacetic acid disodium salt dihydrate. The samples were dehydrated in an ethanol series and embedded in paraffin. Five-micrometer-thickness sections were cut from the root of the first molar in the lower jaw. The sections were stained with hematoxylin and eosin (HE). To evaluate osteoclasts, some sections were stained for tartrate-resistant acid phosphatase (TRAP). TRAP activity was demonstrated using 160 mg/L naphthol AS-BI phosphate (in N,N-dimethyl formamide) as a substrate and 50 mM L(+)-tartaric acid as a coupling agent, which were reacted at 37°C for 40 min.

### 2.4. PDL Cell Isolation

The right and left sides of the experimental samples were considered the occlusal side (Oc side) and non-occlusal side (N-Oc side), respectively. As the PDL was attached to the root surfaces, the first and second mandibular molars were removed to isolate PDL cells. The PDL tissue from both groups was treated with 2 μg/mL collagenase A (Sigma–Aldrich, MO, USA) in phosphate-buffered saline (PBS) for 30 min and 0.1% trypsin (Sigma–Aldrich) in PBS for 10 min at 37°C in a water bath. The PDL cells were cultured in alpha-minimum essential medium (α-MEM; Sigma–Aldrich) containing 10% fetal bovine serum (FBS; Thermo Fisher Scientific, MA, USA) and 1% antibiotic–antimycotic solution (Sigma–Aldrich) at 37°C in an incubator in the presence of 5% CO_2_ for 7–14 days. RAW 264.7 cells were cultured in Dulbecco's modified Eagle's medium (Sigma–Aldrich) containing 10% FBS and 1% antibiotic–antimycotic solution before being cocultured with PDL cells.

### 2.5. Immunocytochemistry

The PDL cells were washed with PBS three times and fixed with 4% paraformaldehyde for 30 min. Permeabilization was performed with 0.3% Triton X-100 in PBS for 5 min, and nonspecific binding was blocked with a blocking buffer (a solution of 1% normal goat serum and 0.1% Tween 20 in PBS) for 60 min. The cells were incubated with monoclonal anti-RANKL (diluted 1:100, GeneTex, CA, USA) and anti-OPG (diluted 1:100, GeneTex) antibodies overnight at 4°C. After washing in PBS, the cells were incubated with Alexa Fluor-conjugated antimouse IgG (diluted 1:500, Life Technologies, OR, USA) antibodies to reveal RANKL and OPG, and counterstained with Hoechst solution (diluted 1:200, Invitrogen, MA, USA). PDL cells were stained with a toluidine blue solution to observe the cell form.

### 2.6. Western Blotting

PDL cells were lysed with 1% radioimmunoprecipitation assay lysis buffer (50 mM Tris-HCl, pH 7.5; 150 mM NaCl; 1% Triton-X 100; 0.1% sodium dodecyl sulfate [SDS]; and 0.5% sodium deoxycholate) in the presence of protease inhibitors. Each sample was separated using SDS–polyacrylamide gel electrophoresis (SDS–PAGE) and blotted onto a polyvinylidene fluoride membrane (Cytiva). After blocking the membrane with a solution of Tris-buffered saline and 0.1% Tween 20 containing 1% nonfat dried milk for 60 min, the membranes were treated with primary anti-RANKL (diluted 1:2000, Proteintech, IL, USA); anti-OPG (diluted 1:2000, GeneTex); and antiglyceraldehyde 3-phosphate dehydrogenase (GAPDH) (diluted 1:2000, Bioss, MA, USA) antibodies, and then incubated with a horseradish peroxidase (HRP)-conjugated secondary antibody (diluted 1:10000, Jackson ImmunoResearch, PA, USA). The enhanced HRP detection reagent EzWestLumi Plus (ATTO, Tokyo, Japan) was used to visualize the stained proteins.

### 2.7. Coculture

As an experimental medium, we supplemented 2 μM L-alanyl–L-glutamine and 284 μM L-ascorbic acid in the aforementioned α-MEM. Experimental cultures were divided into four groups. In the first group, the cells were cultured in α-MEM alone (α-MEM group). In the second group, the cells were maintained with 20 ng/mL soluble recombinant RANKL (sRANKL, Oriental Yeast, Tokyo, Japan) in α-MEM (RANKL group), and in the third group, 20 ng/mL sRANKL and 500 ng/mL anti-OPG were added to the α-MEM (RANKL + anti-OPG group). In the last group, 20 ng/mL sRANKL and 200 ng/mL recombinant OPG (Thermo Fisher Scientific) were added to the experimental medium (RANKL + OPG group). PDL cells (10,000/well) and RAW 264.7 cells (10,000/well) were cocultured in 24-well culture plates. Cultivation was performed for 8 days, and the medium was changed every 3 days. Osteoclast-like cells were stained with TRAP to evaluate osteoclastogenesis.

### 2.8. Statistical Analysis

Assay quantification was performed using three independent experiments with Image-Pro Premier software (Media Cybernetics, MD, USA). In the coculture experiment, we counted and measured the size of the multinucleated TRAP-positive cells (more than three nuclei) in representative areas. The bands obtained by western blotting were scanned, and expression levels of RANKL, OPG, and GAPDH were calculated. The results were presented as the mean value ± standard deviation. All statistical analyses were performed using the Mann–Whitney *U* test, and *p*-values < 0.05 were considered significant.

## 3. Results

### 3.1. Histopathology

**Figure 1:**
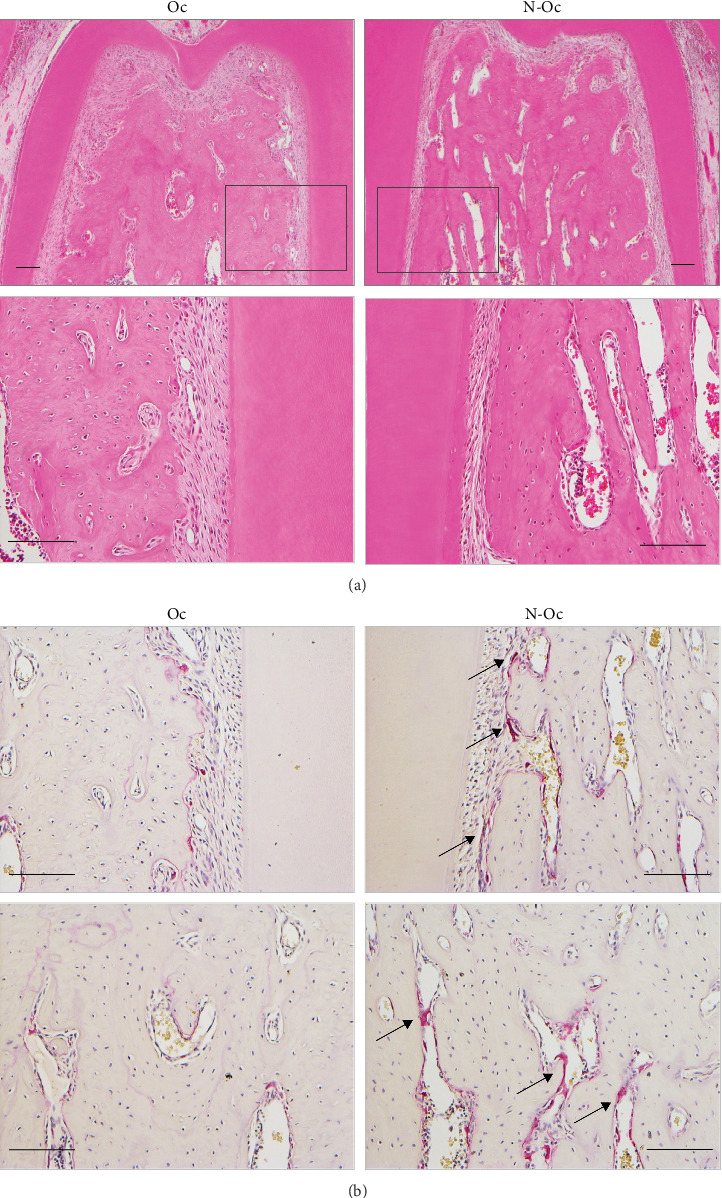
Histological images of the mandible obtained 3 weeks after the extraction of the left maxillary molars. (A) Hematoxylin and eosin staining in the periodontal ligament (PDL) around the furcation, the lower panels show high magnification images of the boxed regions. (B) Tartrate-resistant acid phosphatase (TRAP) staining in the PDL area (upper panels) and marrow space (lower panels). The arrows indicate multinuclear osteoclasts (bar = 100 μm). Oc, occlusal; N-Oc, non-occlusal.

Three weeks after the extraction of the left molars, the PDL on the N-Oc side demonstrated atrophic changes. HE-stained sections showed PDL with narrow spaces and rough or loosely arranged thin periodontal fibers. The number of cells in the PDL also decreased. Furthermore, the marrow space expanded in the interradicular alveolar bone on the N-Oc side. In the TRAP-stained sections, some large osteoclasts were observed on the alveolar bone surface and within the marrow space on the N-Oc side (Figure 1).

### 3.2. Immunocytochemistry

**Figure 2:**
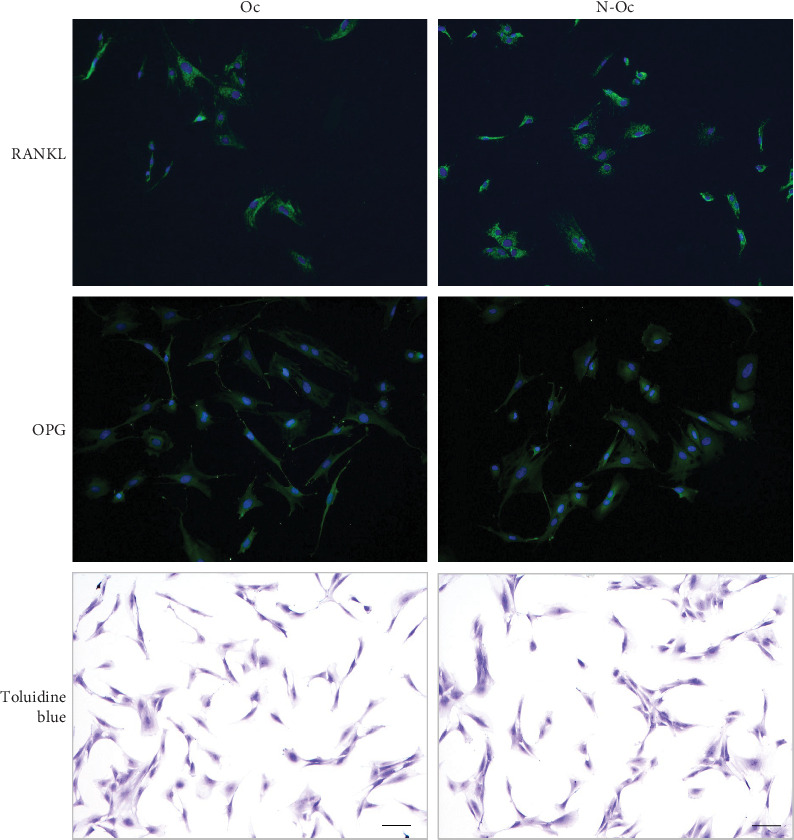
Immunofluorescence analysis and toluidine blue stain in PDL cells. Receptor activator of nuclear factor kappa ligand (RANKL) and osteoprotegerin (OPG) positive cells demonstrate green in immunostaining (bar = 100 μm).

Immunohistochemically, the PDL cells were positive for RANKL and OPG on both sides. There was no difference in the shape or size of PDL cells between the N-Oc and Oc sides (Figure 2).

### 3.3. Western Blotting

**Figure 3:**
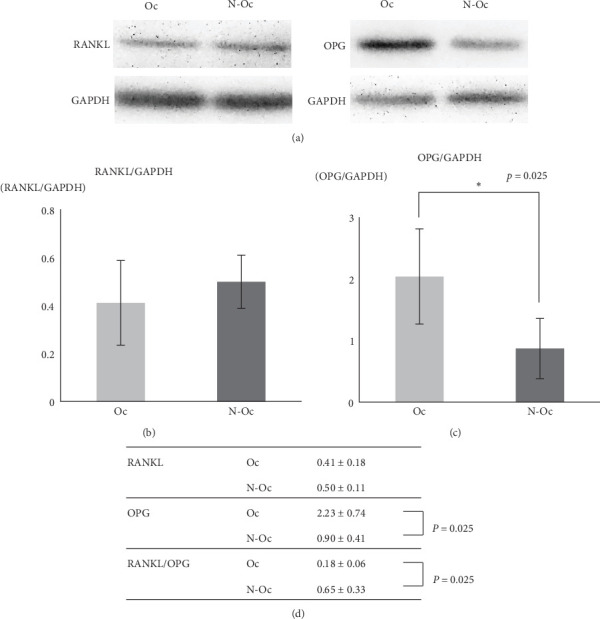
Expression of RANKL, OPG, and glyceraldehyde 3-phosphate dehydrogenase (GAPDH) in PDL cells by western blotting: (A) image of the western blotting, (B) analysis of the RANKL/GAPDH, (C) analysis of the OPG/GAPDH, and (D) ratios of RANKL/GAPDH, OPG/GAPDH, RANKL/OPG expression levels.

RANKL and OPG protein expression in PDL cells were examined by western blotting. Similar RANKL expression levels were observed under both conditions; however, OPG expression was lower on the N-Oc side than on the Oc side (*p*=0.025). The RANKL/OPG expression ratio increased on the N-Oc side significantly (*p*=0.025) (Figure 3).

### 3.4. Coculture

**Figure 4:**
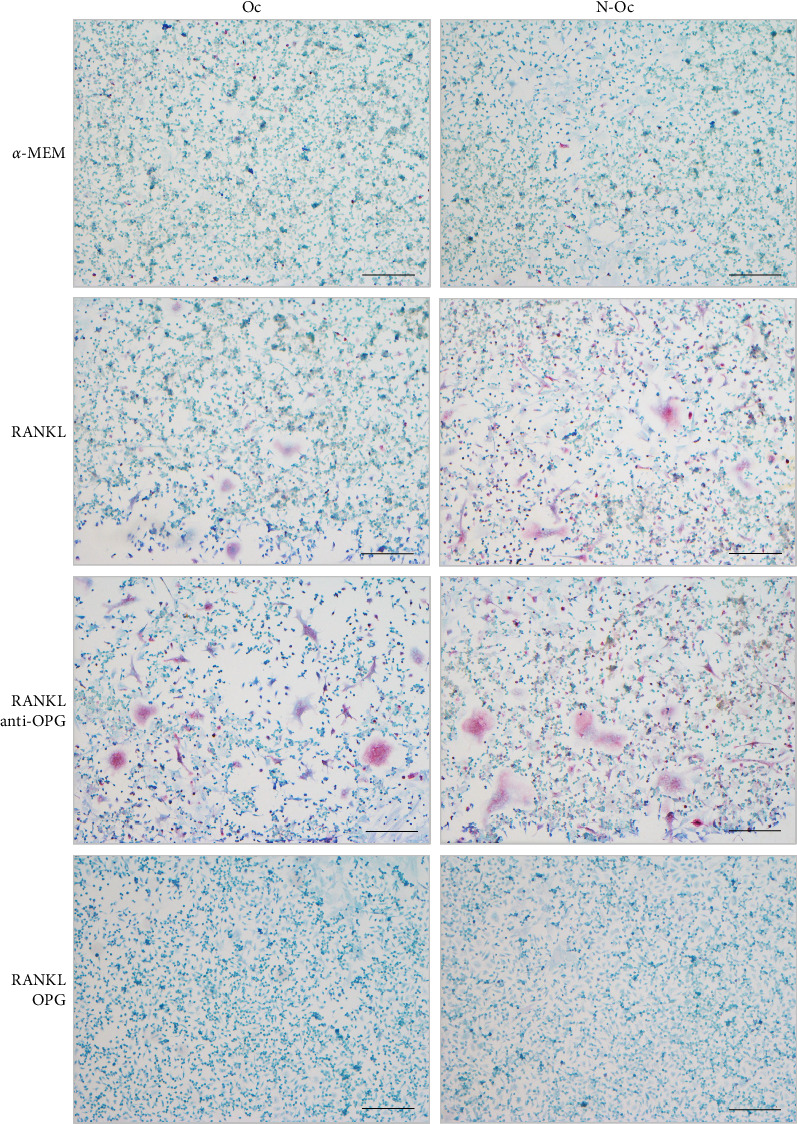
TRAP staining images of cocultured PDL and RAW 264.7 cells. TRAP-positive cells show red color and are counterstained with Giemsa (bar = 100 μm). α-MEM, alpha-minimum essential medium.

In the α-MEM, RANKL, and RANKL + anti-OPG groups, the coculture system for the PDL and RAW 264.7 cells induced the formation of multinuclear osteoclast-like cells, which showed TRAP activity. In the α-MEM group, low numbers of small osteoclast-like cells were observed on both the Oc and N-Oc sides. In the RANKL and RANKL + anti-OPG groups, the number of osteoclast-like cells was higher, and the cells were clearly larger than in the α-MEM group. In the RANKL + OPG group, a few multinuclear osteoclast-like cells were observed (Figure 4).

**Figure 5:**
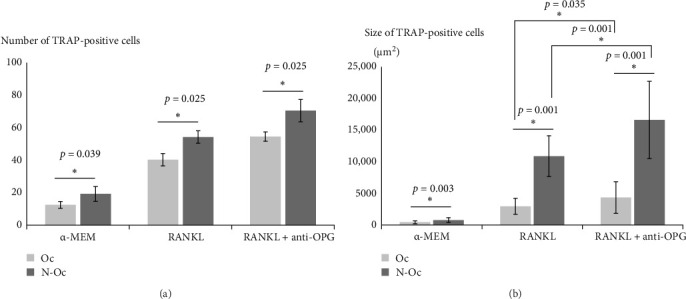
Analysis of the multinucleated TRAP-positive cells: (A) number of TRAP-positive cells and (B) size of TRAP-positive cells.

We analyzed the number and size of TRAP-positive cells. In the α-MEM, RANKL, and RANKL + anti-OPG groups, the number of TRAP-positive cells was significantly higher on the N-Oc side than on the Oc side. The size of the TRAP-positive cells was also significantly larger on the N-Oc side than on the Oc side in the α-MEM, RANKL, and RANKL + anti-OPG groups. Additionally, the size of TRAP-positive cells in the RANKL + anti-OPG group was significantly higher than that in the RANKL group (Figure 5).

## 4. Discussion

Bone remodeling depends on a delicate equilibrium between bone resorption by osteoclasts and bone formation by osteoblasts. Occlusal stimulation plays an important role in the maintenance of alveolar bone and PDL. However, few studies have investigated the role of PDL cells and osteoclast formation in occlusal hypofunction. Furthermore, no in vitro studies have investigated this topic in isolated PDL cells. The present study aimed to elucidate the role of PDL cells in osteoclastogenesis under occlusal hypofunctional conditions.

Occlusal hypofunction was induced by extracting the left molars to eliminate the occlusal force. In this study, the PDL on the N-Oc side showed atrophic changes 3 weeks after molar extraction. Reduced PDL thickness and fewer fibroblasts were observed on the N-Oc side compared to the Oc side. We found some osteoclasts in the alveolar bone near the root in sections subjected to TRAP staining. These findings confirm those of other studies that have reported structural changes in the PDL tissue under occlusal hypofunction conditions [17–19]. Therefore, the approach used to induce occlusal hypofunction in the present study was suitable.

Our data showed that western blotting revealed decreased OPG expression in PDL cells on the N-Oc side. Coculturing RAW 264.7 cells with isolated PDL cells induced the formation of osteoclast-like cells, and the number of TRAP-positive cells on the N-Oc side was significantly higher than that on the Oc side; moreover, the cell size was significantly larger on the N-Oc side than on the Oc side. A comparison of the RANKL + anti-OPG and RANKL groups showed that the size of TRAP-positive cells in the RANKL + anti-OPG group was significantly higher than that in the RANKL group.

Several studies have reported that the intensity and type of mechanical stimulation affect the expression of RANKL and OPG in PDL cells. In an in vitro study, Kanzaki et al. [20] demonstrated that tensile force upregulated RANKL expression in PDL cells. Another study reported that compressive force increases RANKL expression and decreases OPG secretion in PDL cells [21]. There are a few models for examining the effects of reduced mechanical stimulation in isolated PDL cells. Additionally, few studies have reported a relationship between reductions in mechanical stress and the levels of osteoclast-related factors in PDL cells. In an experiment in which osteoblasts were cultured under microgravity conditions, OPG and RANKL expression levels were reduced [22]. Another study showed that the RANKL/OPG expression ratio increased because of a reduction in OPG expression, and greater osteoclast formation was induced by microgravity [23, 24].

In the present study, western blotting revealed decreased OPG expression in PDL cells on the N-Oc side (Figure 3), and such changes may contribute to increased osteoclast activation. This finding suggests that the osteoclastogenesis-related biological functions of mesenchymal cells are affected by a reduction in mechanical stress.

Orthodontic tooth movement is a typical design for in vivo bone remodeling. In clinical and experimental orthodontic treatments, tooth movement causes osteoclast activity on the compression side and osteoblast formation on the tension side, owing to an imbalance between RANKL and OPG expression. Previous studies have shown that orthodontic force increases the number of RANKL-positive cells on the root surface [25] and upregulates the RANKL/OPG ratio to fluctuate more than that of OPG in PDL cells [26]. The regulation of osteoclasts by orthodontic force may affect RANKL rather than OPG, compared to the resulting OPG reduction in this study. Regarding the reduction in mechanical stress, the hindlimbs unloading with tail-suspension is a model of mechanical stress reduction to induce bone disuse, and the enhanced resorptions produce osteoporosis. Hindlimb unloading in mice reportedly results in a change in the expression of RANKL and OPG or their encoding factors in the serum, which is strongly correlated with bone loss [27]. And Dohke et al. [28] reported that hindlimbs unloading revealed regional osteoporotic changes, which were recovered by the resumption of weight bearing on the hind limbs. Therefore, the change following mechanostress unloading is considered to be mostly reversible upon reloading, and the cellular biological potential may also be restored.

Osteoclast formation was induced when PDL cells and supplemental factors were added. In similar coculture experiments using PDL cells, various factors were added to the medium to promote osteoclast formation [6, 29, 30]. Therefore, we attempted to use sRANKL and anti-OPG supplementation of α-MEM to induce osteoclast formation and differentiation. In contrast, recombinant OPG was added to confirm that it inhibited osteoclast formation.

In all experimental groups, the formation of osteoclast-like cells was induced by coculturing RAW 264.7 cells with isolated PDL cells, and we compared the number and size of multinucleated osteoclast-like cells between the groups. When the Oc and N-Oc sides were compared, more osteoclasts were observed on the N-Oc side (Figure 4). These results suggest that PDL cells on the N-Oc side have a greater ability to induce osteoclast formation than those on the Oc side. Subjecting PDL cells to occlusal hypofunction has also been suggested to influence osteoclastogenesis. Our findings agree with those of other studies that reported that under occlusal hypofunction conditions, the alveolar bone was discontinued and bone mineral density was reduced due to increased osteoclast formation [16, 17].

Furthermore, we analyzed the number and size of TRAP-positive cells in each group (Figure 5). The number of TRAP-positive cells on the N-Oc side was significantly higher than that on the Oc side. We showed that PDL cells on the N-Oc side had decreased OPG expression, and that osteoclast activity may have increased. Changes in OPG expression have been reported to affect the regulation of osteoclast differentiation and maturation [31]. We speculate that on the N-Oc side, the number of osteoclasts increased because of an increase in the RANKL/OPG expression ratio caused by a reduction in OPG expression. Regarding cell size, in the RANKL and RANKL + anti-OPG groups, the TRAP-positive cells were significantly larger on the N-Oc side than on the Oc side. Additionally, the comparison of the RANKL + anti-OPG and RANKL groups showed that the TRAP-positive cells in the RANKL + anti-OPG group were significantly larger than those in the RANKL group. In contrast, there was no difference in the number of TRAP-positive cells between the RANKL and RANKL + anti-OPG groups. OPG has been suggested to be involved in the regulation of cell size during osteoclast differentiation. Previous studies have found that osteoclasts were larger in OPG knockout mice than in wild-type mice, and OPG small interfering RNA treatment during osteoclastogenesis enhanced the sizes of osteoclasts [32, 33].

We hypothesized that changes in the RANKL/OPG expression ratio affect the differentiation and maturation of osteoclasts, and that these responses vary depending on the degree of reduction in OPG expression. In this study, we showed that OPG expression in PDL cells decreased on the N-Oc side, and that osteoclast size differed markedly between the RANKL and RANKL + anti-OPG groups. In a coculture of RAW 264.7 cells and PDL cells, the addition of anti-OPG antibodies further reduced OPG activity and increased the RANKL/OPG expression ratio. Changes in osteoclast size may also require an increase in the RANKL/OPG ratio above a certain level. In the RANKL + anti-OPG group, the RANKL/OPG expression ratio on the N-Oc side was appropriate for osteoclast maturation.

Therefore, it is important to discuss the mechanotransduction pathways involved in the regulation of RANKL/OPG expression. Mechanical stress is transduced through several mechanosensors, such as piezochannels and integrins. Piezo1 and Piezo2 are mechanosensors observed in PDL [34], and Piezo1 activity is increased by the mechanical stretching of PDL cells [35]. Furthermore, piezo1 knockdown mice have reduced RANKL expression, which decreases osteoclast numbers [36]. However, little is known about the function of piezo channels in the reduction of mechanical stress, and the response of the PDL to mechanical stress reduction is unclear. Furthermore, osteoclastogenesis in the RANKL/OPG pathway is controlled by Wnt signaling because OPG is a Wnt target gene. The expression level of OPG correlated with Wnt signaling activity. Sclerostin is a Wnt signaling inhibitor of bone remodeling, and the protein expression level of sclerostin increases during occlusal hypofunction [37]. This result may be related to our findings of decreased OPG expression.

A recent study reported that miRNAs are associated with bone remodeling. As one of the most extensively researched miRNAs, miRNA-21 affects both the osteoblastic and osteoclastic processes in bone remodeling. The miRNA-21 has been reported to affect osteoclast differentiation through osteoblast activation in PDL cells [38]. The miRNA-21 deficiency blocks not only bone formation but also bone resorption, and miRNA-21-/- mice show inhibited alveolar osteoclastogenesis compared to wild-type mice [39]. The miRNA-7b directly targets DC-STAMP [40], which is involved in the multinucleation of osteoclasts. In RAW264.7 cells, miRNA-7b overexpression decreased the number of TRAP-positive multinucleated cells, whereas the inhibition of miRNA-7b enhanced osteoclastogenesis [40]. These findings suggest that miRNA-7b controls cell–cell fusion. Some miRNAs expressed in the periodontal tissues, such as miRNA-15, miRNA-17, and miRNA-100, affect bone tissue regulation under inflammatory conditions [41]. It has been implicated in the association between orthodontics and periodontitis. The roles of miRNAs in the regulation of gene expression are widely known, and new miRNAs are constantly being discovered. Thus, miRNAs may have regulated osteoclastogenesis on occlusal hypofunction in this study.

Occlusion-induced mechanical stress is an essential form of stimulation for preserving the periodontal tissue, teeth, and alveolar bone structures. Excessive tooth-related mechanical forces induce occlusal trauma, which is characterized by a reduction in the supporting bone around the periodontal tissue. Our results suggested that occlusal hypofunction promotes bone resorption by increasing the number and activity of osteoclasts. In some animal studies, unloading of the occlusal force by long-term extraction suggested that bone loss occurs in the alveolar bone [42] and around the mesial and distal roots [43]. We propose that long-term occlusal hypofunction increases the risk of alveolar bone loss. Normal occlusion is required for the maintenance of PDL cells by the bone remodeling system, as it ensures an appropriate balance between RANKL and OPG expression.

## 5. Conclusion

Our findings demonstrate that, under occlusal hypofunctional conditions, PDL cells induce changes in their biological functions and promote osteoclast differentiation by reducing OPG expression. Therefore, occlusal stimulation, including the maintenance of alveolar bone, is essential for healthy periodontal tissues.

## Data Availability

The data supporting the findings of this study are available from the corresponding author.
